# Identification of Suitable *Meloidogyne* spp. Housekeeping Genes

**DOI:** 10.21307/jofnem-2019-055

**Published:** 2019-09-20

**Authors:** Weiming Hu, Peter M. DiGennaro

**Affiliations:** 1 Department of Entomology and Nematology, University of Florida, Gainesville FL, 32611

**Keywords:** *Meloidogyne hapla*, *Meloidogyne incognita*, housekeeping gene, Root-knot nematode, RT-qPCR, method, molecular biology, genetics.

## Abstract

Gene expression studies often require reliable housekeeping (HK) genes to accurately capture gene expression levels under given conditions. This is especially true for root-knot nematodes (RKN, *Meloidogyne* spp.), whose drastic developmental changes are strongly dependent upon their environment. Here we utilized a publicly available *M. hapla* RNAseq database to identify putative HK genes throughout the nematode lifecycle. We then validated these candidate HK genes on *M. incognita* in order to develop a small library of suitable HK genes for RKN. Seven putative HK genes were selected for validation based on high expression level and ease of primer design. The expression of these genes was quantified by qPCR at different developmental stages to capture the entire life cycle of *M. incognita* which included eggs and naive infective juveniles through 3-wk post inoculation. Two algorithms, geNorm and Normfinder, identified three genes (*Disu*, *Poly*, and *Skinase*) constitutively and uniformly expressed throughout the entire life cycle of RKN. We believe these genes are superior HK genes suitable to be used as internal reference genes at all stages of RKN. Importantly, while we identified *Actin*, a commonly used HK gene, as a candidate gene within our RNAseq analyses, our qPCR results did not demonstrate stable expression throughout the nematode life cycle of this gene. This study successfully validated suitable HK genes utilizing both RNAseq data and standard qPCR methods across two species of RKN; suitable HK genes are likely applicable to other species of RKN, or even plant-parasitic nematodes. Additional lists of potential HK genes are also provided if the nematode of interest does not have homologues of the three superior reference genes described here. Gene expression studies on RKN should use validated HK genes to ensure accurate representation of transcript abundance.

Root-knot nematodes (RKN, *Meloidogyne* spp.) are sedentary endoparasites with wide host ranges, including most important agricultural crops, and cause billions of dollars in yield losses (Sasser and Freckman, 1987). The first genomes of *M. hapla* and *M. incognita* were available in 2008 (Abad et al., 2008; Opperman et al., 2008), and now the genomes of seven RKN species, including the abovementioned two, and *M. arenaria*, *M. enterolobii*, *M. floridensis*, *M. javanica* are publicly available. The emergence of available genomes and transcriptomes are enabling the discovery of plant–nematode interaction mechanisms and nematode loci involved in parasitism (Gleason et al., 2017). Expression of nematode genes is an important factor in determining a role in parasitism. However, housekeeping (HK) genes for RKN have not been defined and validated, reducing the accuracy and credibility of gene expression studies.

Reverse transcription quantitative PCR (RT-qPCR) is a standard for quantifying mRNA because of its high sensitivity and flexibility (Bustin, 2000). Advantages of RT-qPCR include accurate quantification, flexible wide range, potential high throughput, and wide application in different types of samples (Huggett et al., 2005). However, the reliability of RT-qPCR depends on normalization to control variations such as quantity and quality of RNA in the starting material, efficiency of cDNA transcription synthesis, and pipetting during any of the process from RNA preparation to qPCR (Gal et al., 2006). Generating standard curves for target genes is a common way to quantify gene expression, but with thousands of putative parasitism genes within RKN genomes, it is impractical to produce a unique standard curve for every gene of interest. An alternative approach is to use HK genes which are stably expressed regardless of the given conditions.

HK genes are genes necessary to maintain basic cellular function and are presumed to be expressed in all cells of organisms (Butte et al., 2001). HK genes are often used as endogenous references to normalize the variations in RT-qPCR experiments (Jian et al., 2008). However, under conditions such as heat stress, viral infection, and cancer development, the expression of HK genes of animals can vary (Sahu et al., 2018). The utilization of invalidated reference genes results in unreliable and misleading conclusions. There is an urgent need to discover and validate suitable HK genes for plant-parasitic nematodes to ensure accurate representation of RKN transcript abundance.

Commonly used HK genes in plants and animals are tubulins, actins, glyceraldehyde-3- phosphate dehydrogenase (*GAPDH*), albumins, cyclophilin, micro-globulins, ribosomal units (18S or 28S rRNA), ubiquitin (*UBQ*), and elongation factors (de Jesus Miranda et al., 2013). Suitable HK genes of plants such as *Arabidopsis* (Hofmann and Grundler, 2007), tomato (Exposito-Rodriguez et al., 2008), and soybean (de Jesus Miranda et al., 2013) have been discovered and validated using RNAseq and RT-qPCR. However, there is a huge gap in defining and identifying superior HK genes as internal reference genes in RKN, even though the genome of various species of which has been assembled and annotated. Without validation, the detection of “parasitism” gene expression differences during nematode development is hardly convincing (Duarte et al., 2016).

This study utilizes a publicly available *M. hapla* RNAseq database to identify putative HK genes suitable throughout the RKN life cycle. We validated across a select number of these candidate HK genes with a second species of RKN, *M. incognita*, using standard RT-qPCR, demonstrating that the HK genes provided in this study could apply to other species of RKN. Importantly, we discovered that the widely used HK gene-*Actin* may not be suitable reference gene throughout the life cycle of RKN. This study serves as a stepping stone for gene expression studies of RKN, and can greatly facilitate transcriptomic studies of plant-parasitic nematode.

## Materials and methods

### Candidate HK gene selection

We utilized the publicly available *M. hapla* RNAseq database (https://brcwebportal.cos.ncsu.edu/haplatome/1-0_hapla.php) to discover potential HK genes for RKN (Cha, 2016). Genes with more than five reads per sample across all the treatments were analyzed. Differently expressed genes (DEG) were determined in R v.3.3.3 (Team, 2006) using package “edgeR” (McCarthy et al., 2012). Specifically, the count of the genes was normalized using function “calcNormFactors”, and then the dispersion was estimated using function “estimateDisp”; finally, pairwise comparison was accomplished using function “exactTest”. Genes with comparision value 0 across all treatments were considered non-DEG. The non-DEG with matching loci within the *M. hapla* genome were subject to SignalP 4.1 to determine whether they encode secreted proteins ( Petersen et al., 2011), putative secreted protines were omitted as candidates due to the possibility of direct involvement in the plant–nematode interaction. The details of candidate HK genes are in listed in Table A1. The gene functions were annotated utilizing both KEGG (Kanehisa and Goto, 2000) and WormBase parasite website (Howe et al., 2017). Seven genes of the non-DEG pool were selected based upon ease of primer design and high expression level to validate stability of gene expression throughout the life cycle of *M. incognita* in growth chamber assays.

**Table A1.** List of stably expressed genes in *M. hapla* based on RNAseq database.

*M. hapla* gene ID	Annotation
MhA1_Contig20.frz3.gene50	Intramembane protease 2
MhA1_Contig609.frz3.gene1	Not annotated
MhA1_Contig627.frz3.gene3	Fructose-bisphosphate aldolase 2
MhA1_Contig649.frz3.gene8	Myosin regulatory light chain 2
MhA1_Contig675.frz3.gene6	Probable arginine kinase
MhA1_Contig684.frz3.gene7	Probable splicing factor, arginine/serine-rich 5
MhA1_Contig91.frz3.gene33	elongation factor 1-alpha
MhA1_Contig95.frz3.gene17	elongation factor 1-alpha
MhA1_Contig96.frz3.gene30	Troponin 1
MhA1_Contig113.frz3.gene12	A-kinase anchor protein 1, mitochondrial
MhA1_Contig113.frz3.gene45	Heat shock 70 kDa protein
MhA1_Contig1167.frz3.gene2	KIN-4 protein
MhA1_Contig130.frz3.gene58	not annotated
MhA1_Contig131.frz3.gene10	40 S ribosomal protein S6
MhA1_Contig1354.frz3.gene2	not annotated
MhA1_Contig163.frz3.gene44	not annotated
MhA1_Contig1687.frz3.gene1	not annotated
MhA1_Contig1972.frz3.gene3	Heat shock protein 90
MhA1_Contig2015.frz3.gene6	Bax inhibitor 1
MhA1_Contig2458.frz3.gene1	not annotated
MhA1_Contig253.frz3.gene11	not annotated
MhA1_Contig253.frz3.gene39	not annotated
MhA1_Contig256.frz3.gene21	Vinculin
MhA1_Contig261.frz3.fgene1	Dynein heavy chain, cytoplasmic
MhA1_Contig342.frz3.gene52	Activating signal cointegrator 1 complex subunit 3
MhA1_Contig349.frz3.gene33	spliceosime RNA helicae DDX39B homolog
MhA1_Contig400.frz3.gene18	FACT complex subunit SSRP1
MhA1_Contig800.frz3.gene34	Calsyntenin-1
MhA1_Contig1000.frz3.gene28	60 S ribosomal protwin L3
MhA1_Contig1372.frz3.fgene1	Glutathione S-transferase P
MhA1_Contig2493.frz3.fgene1	Not annotated

### Plant and nematode inoculation

To test whether the genes can be widely applied to all RKN, a growth chamber experiment was set up to validate candidate HK genes on *M. incognita*. Tomato seeds (variety “Rutgers”) were surface sterilized and planted in potting soil. After 3 wk, seedlings were transferred into individual pots with sand. Second stage juveniles (J2) of *M. incognita* were hatched from freshly collected eggs, and 300 J2 were inoculated into each pot. The pots were kept in a growth room with 16 hr light/8 hr dark at 26°C. The freshly collected eggs and J2 were also stored in −80°C for RNA extraction.

### Root sample collection

Roots were gently washed in sterile water, dried and immediately frozen in liquid nitrogen. A total of 11 time points were collected with five time points during early infection, (1, 2, 4, 5, and 7 days post inoculation-dpi) and six time points during nematode maturity (21 dpi) (Table 1). To test diurnal effects on RKN gene expression, at the nematode maturity stage, root samples were collected three time points in dark and three time points in light (Table 1). Each of the total 11 time points had four biological replicates, and 3-wk old non-infected tomato plants were used as negative control.

**Table 1.** Root sampling time points.

First week	Three weeks dpi^a^
1 dpi	*Light*
2 dpi	05:30
4 dpi	13:00
5 dpi	20:00
7 dpi	*Dark*
	21:30
	23:00
	04:00

^a^dpi: day post inoculation; The light was turned on at 05:00 and turned off at 21:00 in the growth room daily.

### Primer design

The homologues of *M. hapla* HK gene of *M. incognita* were obtained through WormBase parasite website. The transcript sequences of *M. incognita* V3 were used as template in IDT primerQuest tool for RT-qPCR primer design with the default parameters. To avoid any possible DNA amplification which would alter transcript level detection, primers were designed to span an exon–exon junction if possible or target adjacent exons. We checked the specificity of the primers against the whole transcriptome of *M. incognita* using Primer-BLAST on the NCBI website. The corresponding gene ID and annotation of the candidate HK genes are included in Table 2, and two genes with high to low DEG value, plus a known actin gene (Duarte et al., 2016) were used as controls (Table A2).

**Table 2.** Validated Candidate housekeeping genes and primers for *M. incognita.*

Gene symbol	Forward	Reverse	*M. hapla* gene ID	*M. incognita* homologue loci	Annotation
*Actin1*	GGGTATGGAATCTGCTGGTATT	CATGGTCGTACCTCCAGAAAG	MhA1_Contig4.frz3.gene2	Minc3s00730g16611 Minc3s00960g19311	Actin
*Disu*	CACCGCTAATGAAGTTGAAGATG	CGCTCTCCAGTGTAGTCAATAA	MhA1_Contig165.frz3.gene9	Minc3s01268g22290 Minc3s02350g29607 Minc3s04154g35616	protein disulfide-isomerase
*Minc04440*	GCCGCCATCTTGGCTATTA	GGTGGGACCAAATCCTTGTAT	MhA1_Contig113.frz3.gene11	Minc3s00096g04440 Minc3s00113g04971 Minc3s00259g08816	not annotated
*Poly*	ACGTCCAGAGAATCTGACAAAC	CCACGGCAATAGCGAGATAC	MhA1_Contig123.frz3.gene87	Minc3s01682g25559 Minc3s03281g33396	Polyadenylate-binding protein
*ELF*	CTCCTGGACACGTTGACTTC	GCACACAAACTCCTGAAACAC	MhA1_Contig256.frz3.fgene4	Minc3s00468g12850 Minc3s00594g14836 Minc3s01848g26725 Minc3s02628g30935	Elongation factor 2
*PTP*	GGAAAGCATTTTCACCTGCGA	ACGGGTTGCCAACAGATGAA	MhA1_Contig342.frz3.gene42	Minc3s00073g03598 Minc3s00981g19546 Minc3s03830g34926	protein transport
*Skinase*	AGCGTTTGGTTGCTCTCTCA	GACTCTCCCACGCAAGGAAT	MhA1_Contig647.frz3.gene12	Minc3s00176g06827 Minc3s00279g09289 Minc3s03755g34706 Minc3s09595g43359 Minc3s11722g45107	Serine/threonine-protein kinase smg-1

**Table A2.** Primers and annotation for differently expressed genes.

Gene name	Forward	Reverse	*M. hapla* gene ID	*M. incognita* gene ID	Annotation
*Colla*	CCAATCTCTCTACAGGCAGAAAC	GGACCAGGTGCGCAATTA	MhA1_Contig1040.frz3.gene14	Minc3s00036g02116 Minc3s00100g04559	nematode cuticle collagen
*NHL*	GTCCCCACAGGCACTTTCTT	GCCGACGAGGAAGTGTTCTT	MhA1_Contig7.frz3.gene17	Minc3s00018g01178 Minc3s00027g01714	NHL repeat
*Actin2*	GATGGCTACAGCTGCTTCGT	GGACAGTGTTGGCGTAAAGG			

### RNA extraction and cDNA synthesis

RNA was extracted from the root samples, eggs and naïve, pre-parasitic J2 using RNeasy Plant Mini kit (Qiagen, USA) according to the manufacturer’s protocol. cDNA was synthesized with the iScript Reverse Transcription Supermix (Bio-RAD) for RT-qPCR according to the manufacturer’s instructions.

### RT-qPCR

RT-qPCR was carried out with a StepOne Plus Real Time PCR detection system (Applied Biosystem, USA) using SYBR Green as detection agent. PCR reactions were conducted with three technical replications for each sample in MicroAmp Fast 96 well reaction plate (0.1 ml) (Applied Biosystems). The total volume of the reaction was 10 μl with 0.5 μl forward and reverse primers each at 10 μM, 2.5 μl cDNA template, 1.5 μl of Nuclease free water and 5 μl SYBR Green qPCR MasterMix (iTaq Universal SYBR Green Supermix, Bio-RAD). PCR was performed at 95°C for 20 sec followed by 40 cycles at 95°C for 3 sec, and 60°C for 30 sec, the melting curve was generated at 95°C for 15 sec, 60°C for 1 min, and 95°C for 15 sec. Ct value was calculated using the StepOne software V2.3 (Applied Biosystems).

### Data analyses

To ensure no off-target amplicons were produced, the melt curve was checked carefully to only include samples with single peaks at the correct melting temperature for downstream analyses. The mean C_T_ (cycle threshold) value of each sample was calculated in excel from three technical replicates. The data were organized in excel according to the suggested format by NormFinder (Jensen and Ørntoft, 2004). According to the algorithm of NormFinder, candidate HK genes were compared pairwise within and across groups resulting in a stability value; the most stable HK genes are those with the lowest stability value. In our study, each time point was treated as a group factor. To test the robust of NormFinder analyses, the *C*_T_ value was also submitted to geNorm, another similar HK gene algorithm, for stability calculation (Vandesompele et al., 2002).

## Results

### Functional summary of candidate HK genes

**Figure 1:**
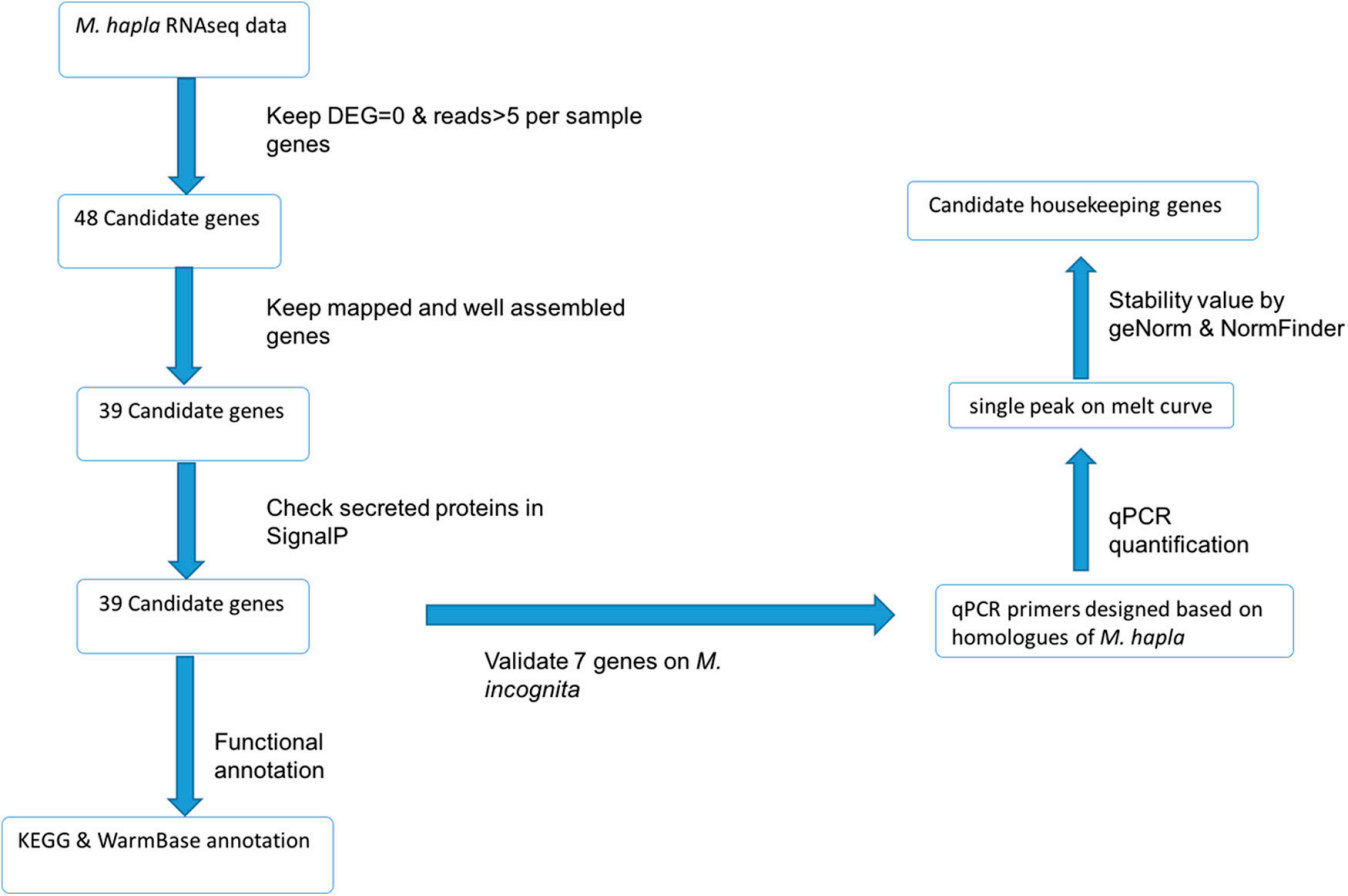
Candidate housekeeping gene selection pipeline.

**Figure 2:**
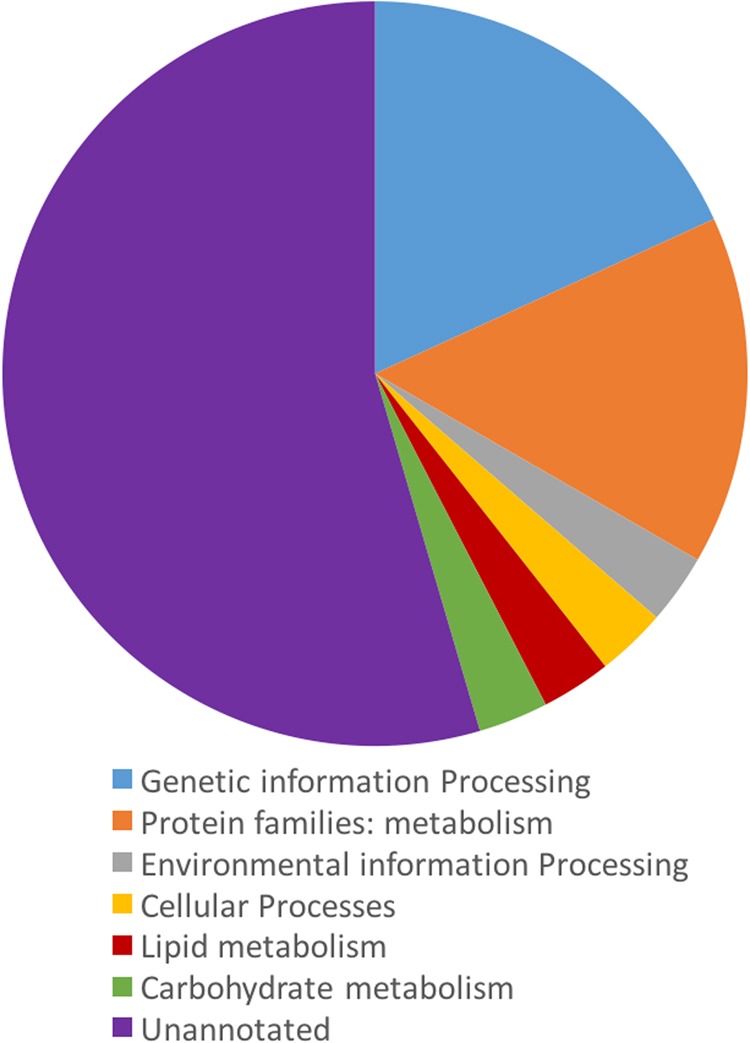
Summary of KEGG annotation of the candidate housekeeping genes.

Candidate HK genes were identified utilizing RNAseq database and then annotated with KEGG and WarmBase parasite. After filtering out genes with low count and DEG >0, we identified 48 candidate HK genes (Fig. 1). While only 39 out of the 48 genes were mapped to *M. hapla* genome, the 39 genes did not have the potential to encode secreted protein (Fig. 1) and are less likely to be directly involved in the plant–nematode interaction. The remaining 39 genes were submitted to KEGG for functional annotation; only 15 out of 39 genes were annotated (Fig. 2), and majority of the genes belong to genetic information processing and metabolism (Fig. 2). The annotation incorporated the results of KEGG and Wormbase was listed in Table A1.

### HK gene validation

Seven out of the 39 candidate HK genes were validated in *M. incognita* based on high expression level and ease of primer design. Primer specificity was confirmed by single peak on the melt curve. Uninfected tomato plant root samples were not amplified by the primer sets, indicating that all primers were specific to RKN. We used two algorithms to validate suitable HK genes through all developmental stages, including a 24 hr period at 3-wk post inoculation. We determined genes with low stability values as stably expressed genes. Gene stability is slightly different between the two algorithms and throughout the life cycle of RKN.

**Figure 3:**
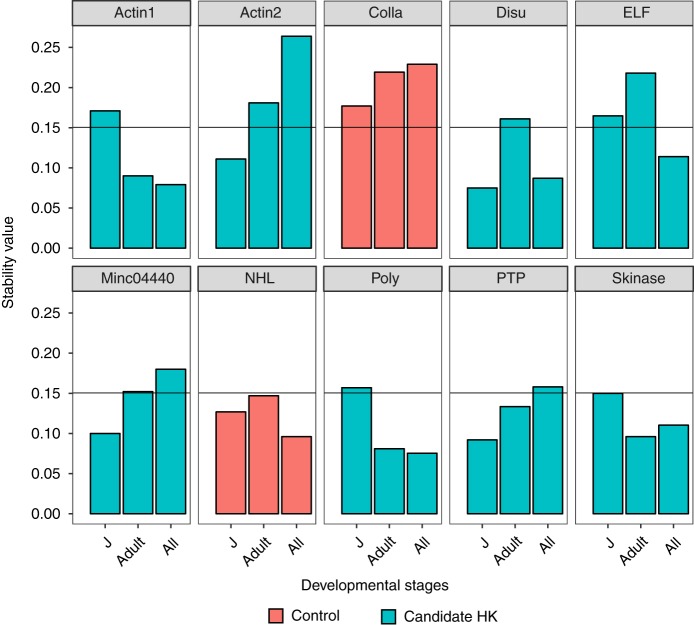
Stability value of validated housekeeping genes across life cycle of *M. incognita* calculated by geNorm. Juvenile stage samples were collected within the first week, adult stage samples were collected 3 wk after inoculation, and all stages samples include previous stages, eggs and uninfected juveniles. Orange bars are negative control which were differently expressed genes (Colla: DEG = 10; NHL: DEG = 1), and blue bars are candidate housekeeping genes. Genes with stability value below 0.15 are considered suitable reference genes.

**Figure 4:**
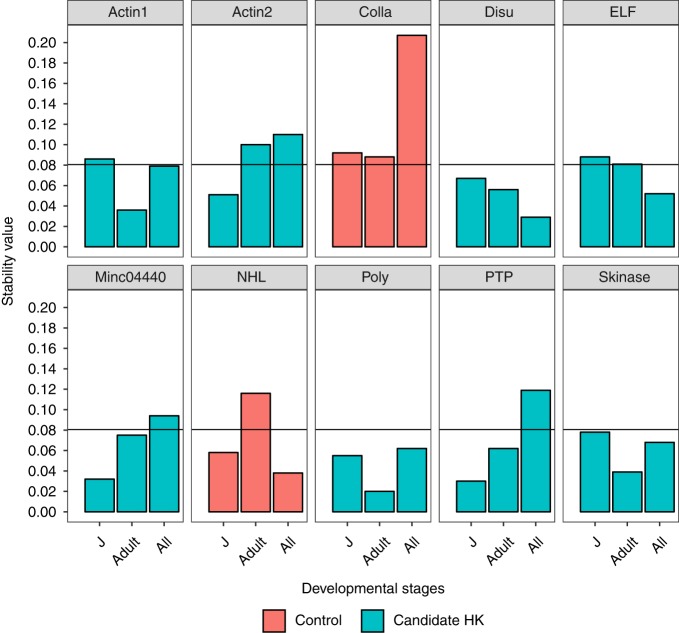
Stability value of validated housekeeping genes across life cycle of *M. incognita* calculated by NormFinder. Juvenile stage samples were collected within the first week, adult stage samples were collected 3 wk after inoculation, and all stages samples include previous stages, eggs and uninfected juveniles. Orange bars are negative control which were differently expressed genes (Colla: DEG = 10; NHL: DEG = 1), and blue bars are candidate housekeeping genes. Genes with stability value below 0.08 are considered suitable reference genes for this study.

The geNorm algorithm defines any gene with stability value less than 0.15 as stable genes; RKN genes under the cut-off value were selected as HK genes. According to geNorm, *Actin1*, *Disu*, *ELF*, *Poly*, *Skinase* were all suitable HK genes throughout all the developmental stages of RKN (Fig. 3). However, for naïve juveniles, *Actin2*, *Disu*, *Minc04440*, *PTP*, and *Skinase* were better reference genes for transcript quantification (Fig. 3). In addition, over a 24 hr period (diurnal) at the adult stage, *Actin1*, *ELF*, *Poly*, *Skinase* were determined suitable HK genes (Fig. 3). In summary, *Disu, Poly*, and *Skinase* had stability values below the threshold for any developmental stage. Normfinder yielded similar yet slightly different results than geNorm. When including all stages, *Disu*, *ELF*, *Poly*, *Skinase* were suitable reference genes (Fig. 4), but *Actin2*, *Disu*, *Minc04440*, *Poly*, *PTP*, and *Skinase* were stably expressed gene at naïve juvenile stage (Fig. 4). Lastly, *Disu*, *ELF*, *Poly*, and *Skinase* were better HK genes at the mature stage over the 24 hr period (Fig. 4). Overall, *Disu*, *Poly*, and *Skinase* were suitable HK genes for any developmental stage as suggested by Normfinder. Importantly, our negative control gene, *Colla*, was highly variable based on RNAseq data, and was also determined to be highly variably expressed throughout all developmental stages by both algorithms.

## Discussion

Gene expression is in a new era with the constant advancement of next generation sequencing. The expression of thousands of genes can be evaluated in a single experiment, but it is impossible to validate the expression of these genes through standard qPCR by generating standard curves. Reliable and suitable HK genes are essential for reporting accurate gene expression; however, there are no validated HK genes for RKN. The commonly used HK genes, such as 16S and *Actin*, are frequently used, yet lack any experimental support (Gleason et al., 2017). Although Actin was one of the first HK genes to be used as a normalizer, transcript levels have been demonstrated to vary widely under experimental conditions in human breast epithelial cells, blastomeres, porcine tissues, and canine myocardium (Bustin, 2000). Our results also indicate that *Actin* might not be a suitable HK gene RKN (Figs. 3,4). Ribosomal 16S/18S rRNA are useful internal control genes, but rRNA abnundance is much higher than target mRNA transcript levels, which makes pairwise statistical analyses inaccurate (Bustin, 2000). We successfully identified and validated the most suitable HK genes throughout the life cycle of RKN: *Disu*, *Poly*, and *Skinase*. These three genes are constitutively and uniformly expressed at all stages of RKN development and can be widely used as reference genes to quantify relative gene expression levels. Importantly, the homologs these genes can be found in over 150 nematode species. The HK genes for a specific species can be easily secured by looking up the homologs of those two genes, as we tested that they were stably expressed across *M. hapla* and *M. incognita*. We also provided a list of candidate HK genes to narrow down the search of HK genes if the homologs of the validated HK genes do not exist in a nematode. Thus, the work presented here can be easily adapted to discover HK genes for other nematodes of interest.

We used two standard algorithms to validate putative HK genes identified in the RNAseq analyses to ensure reliable results. Both algorithms produced consistent results, suggesting that Normfinder and geNorm are robust programs for finding HK genes. In fact, those two programs have been used widely to determine HK genes in human, animals, and plants (Czechowski et al., 2005; Jian et al., 2008; Jongsik et al., 2007; Sahu et al., 2018). The stability value of our candidate HK genes were much lower than those in plants (Czechowski et al., 2005), suggesting that the candidate genes were stably expressed. The threshold of Normfinder was not suggested by the developer, but in this specific study, a similar pattern was observed if the cut-off value was 0.08. It is largely agreed that a single HK gene is not suitable to normalize expression data; at least three HK genes are suggested by geNorm (Vandesompele et al., 2002). Overall, both programs suggested that *Disu*, *Skinase*, and *Poly* are suitable HK genes at any developmental stage of RKN. We also suggest that genes with lower stability value should be chosen if only quantifying the expression of genes in a specific stage of the life cycle. Such as only for naïve juvenile stage, *Disu*, *Minc04440*, and *PTP* are great HK genes, while at the adult stage, *Poly*, *PTP*, and *Skinase* are better options (Figs. 3,4). Similar phenomena were observed in human cells and plants when certain HK genes are not suitable for experimental condition. Some HK genes constitutively expressed only in a particular tissue, and only in fetal or adult in human (Butte et al., 2001). The potential HK genes of *Arabidopsis thaliana* were tested on various tissues and under different biotic conditions to ensure the consistency (Czechowski et al., 2005). Interestingly, widely used HK genes such as *UBQ10*, *ACT2*, and *EF1a* for *Arabidopsis thaliana* were not stable when infected with RKN or soybean cyst nematode (Hofmann and Grundler, 2007). In addition, different combination of HK genes were also suggested for the tomato development process (Exposito-Rodriguez et al., 2008).

The expression level of the gene is highly related with its function during the life cycle. The RKN nematode gene *Colla* is involved in the nematode cuticle collagen process and was expected to be highly variable during the life cycle of RKN. RNAseq analyses (DEG = 10) and standard qPCR results (Figs. 3,4) demonstrated highly variable expression in naïve juvenile, adult, and all stages of the life cycle, as expected for a cuticular collagen gene. Another negative control gene- NHL- with DEG value 1, is an amino acid repeat found in many enzymes. RNAseq data of this gene suggested that it was only differently expressed between egg and non-infected juvenile (Cha, 2016), which coincided with our results that it only expressed at the mature stage when eggs are formed, and naïve juveniles were hatched (Fig. 4). We deduce that genes involved in encoding disulfide-isomerase (*Disu*), Polyadenylate-binding protein (*Poly*), protein transport (*PTP*), and Serine/threonine-protein kinase (*Skinase*) are good HK genes for plant-parasitic nematodes as these are loci involved in processes required throughout the lifecycle of the nematode.

## References

[ref001] Abad P., Gouzy J., Aury J. M., Castagnone-Sereno P., Danchin E. G. J., Deleury E., Perfus-Barbeoch L., Anthouard V., Artiguenave F., Blok V. C., Caillaud M. C., Coutinho P. M., Dasilva C., De Luca F., Deau F., Esquibet M., Flutre T., Goldstone J. V., Hamamouch N., Hewezi T., Jaillon O., Jubin C., Leonetti P., Magliano M., Maier T. R., Markov G. V., McVeigh P., Pesole G., Poulain J., Robinson-Rechavi M., Sallet E., Segurens B., Steinbach D., Tytgat T., Ugarte E., van Ghelder C., Veronico P., Baum T. J., Blaxter M., Bleve-Zacheo T., Davis E. L., Ewbank J. J., Favery B., Grenier E., Henrissat B., Jones J. T., Laudet V., Maule A. G., Quesneville H., Rosso M. N., Schiex T., Smant G., Weissenbach J. and Wincker P. 2008 Genome sequence of the metazoan plant-parasitic nematode *Meloidogyne incognita*. Nature Biotechnology 26:909–15. 10.1038/nbt.1482 18660804

[ref002] Bustin S. A. 2000 Absolute quantification of mRNA using real-time reverse transcription polymerase chain reaction assays. Journal of Molecular Endocrinology 25:169–93. 11013345 10.1677/jme.0.0250169

[ref003] Butte A. J., Dzau V. J. and Glueck S. B. 2001 Further defining housekeeping, or ‘maintenance’, genes focus on ‘a compendium of gene expression in normal human tissues’. Physiological Genomics 7:95–6. 11773595 10.1152/physiolgenomics.2001.7.2.95

[ref004] Cha S. 2016 Is the biology of M. chitwoodi and M. hapla reflected in their genomes? North Carolina State University, Raleigh, NC.

[ref023] Czechowski T. 2005 Genome-wide identification and testing of superior reference genes for transcript normalization in Arabidopsis. Plant physiology. 139 (1):5–17. 16166256 10.1104/pp.105.063743 PMC1203353

[ref005] de Jesus Miranda V., Coelho R. R., Viana A. A. B., de Oliveira Neto O. B., Carneiro R. M. D. G., Rocha T. L., de Sa M. F. G. and Fragoso R. R. 2013 Validation of reference genes aiming accurate normalization of qPCR data in soybean upon nematode parasitism and insect attack. BMC Research Notes 6:196. 23668315 10.1186/1756-0500-6-196 PMC3660166

[ref006] Duarte A., Maleita C., Tiago I., Curtis R. and Abrantes I. 2016 Molecular characterization of putative parasitism genes in the plant-parasitic nematode *Meloidogyne hispanica*. Journal of Helminthology 90:28–38. 25319213 10.1017/S0022149X1400073X

[ref007] Exposito-Rodriguez M., Borges A. A., Borges-Perez A. and Perez J. A. 2008 Selection of internal control genes for quantitative real-time RT-PCR studies during tomato development process. BMC Plant Biology 8 (1):131. 19102748 10.1186/1471-2229-8-131 PMC2629474

[ref008] Gal A. B., Carnwath J. W., Dinnyes A., Herrmann D., Niemann H. and Wrenzycki C. 2006 Comparison of real-time polymerase chain reaction and end-point polymerase chain reaction for the analysis of gene expression in preimplantation embryos. Reproduction Fertility and Development 18:365–71. 10.1071/rd05012 16554012

[ref009] Gleason C., Polzin F., Habash S. S., Zhang L., Utermark J., Grundler F. M. W. and Elashry A. 2017 Identification of two *Meloidogyne hapla* genes and an investigation of their roles in the plant–nematode interaction. Molecular Plant–Microbe Interactions 30:101–12. 28301312 10.1094/MPMI-06-16-0107-R

[ref010] Hofmann J. and Grundler F. M. W. 2007 Identification of reference genes for qRT-PCR studies of gene expression in giant cells and syncytia induced in Arabidopsis thaliana by *Meloidogyne incognita* and *Heterodera schachtii*. Nematology 9:317–23.

[ref011] Howe K. L., Bolt B. J., Shafie M., Kersey P. and Berriman M. 2017 WormBase ParaSite - a comprehensive resource for helminth genomics. Molecular and Biochemical Parasitology 215:2–10. 27899279 10.1016/j.molbiopara.2016.11.005 PMC5486357

[ref012] Huggett J., Dheda K., Bustin S. and Zumla A. 2005 Real-time RT-PCR normalisation; strategies and considerations. Genes and Immunity 6:279–84. 15815687 10.1038/sj.gene.6364190

[ref013] Jensen J. and Ørntoft T. 2004 Normalization of real-time quantitative RT-PCR data: a model based variance estimation approach to identify genes suited for normalization-applied to bladder-and colon-cancer data-sets. Cancer Research 64:5245–50. 15289330 10.1158/0008-5472.CAN-04-0496

[ref014] Jian B., Liu B., Bi Y. R., Hou W. S., Wu C. X. and Han T. F. 2008 Validation of internal control for gene expression study in soybean by quantitative real-time PCR. BMC Molecular Biology, 9. 18573215 10.1186/1471-2199-9-59 PMC2443375

[ref015] Jongsik C. 2007 EzTaxon: a web-based tool for the identification of prokaryotes based on 16S ribosomal RNA gene sequences. International journal of systematic and evolutionary microbiology 57 (10):2259–61. 17911292 10.1099/ijs.0.64915-0

[ref016] Kanehisa M. and Goto S. 2000 KEGG: Kyoto encyclopedia of genes and genomes. Nucleic Acids Research 28:27–30. 10592173 10.1093/nar/28.1.27 PMC102409

[ref017] McCarthy D. J., Chen Y. S. and Smyth G. K. 2012 Differential expression analysis of multifactor RNA-Seq experiments with respect to biological variation. Nucleic Acids Research 40:4288–97. 22287627 10.1093/nar/gks042 PMC3378882

[ref018] Opperman C. H., Bird D. M., Williamson V. M., Rokhsar D. S., Burke M., Cohn J., Cromer J., Diener S., Gajan J. and Graham S. 2008 Sequence and genetic map of Meloidogyne hapla: A compact nematode genome for plant parasitism. Proceedings of the National Academy of Sciences 105:14802–7. 10.1073/pnas.0805946105 PMC2547418 18809916

[ref019] Petersen T. N., Brunak S., von Heijne G. and Nielsen H. 2011 SignalP 4.0: discriminating signal peptides from transmembrane regions. Nature Methods 8:785–6. 21959131 10.1038/nmeth.1701

[ref020] Sahu A. R., Wani S. A., Saxena S., Rajak K. K., Chaudhary D., Sahoo A. P., Khanduri A., Pandey A., Mondal P., Malla W. A., Khan R. I. N., Tiwari A. K., Mishra B., Muthuchelvan D., Mishra B. P., Singh R. K. and Gandham R. K. 2018 Selection and validation of suitable reference genes for qPCR gene expression analysis in goats and sheep under Peste des petits ruminants virus (PPRV), lineage IV infection. Scientific reports 8. 10.1038/s41598-018-34236-7 PMC6206032 30374051

[ref021] Sasser J. and Freckman D. 1987 W 1987 A world perspective on nematology. The role of the society. Vistas on Nematology a Commemoration of the Twenty Fifth Anniversary M A Siddiqai and MM Alam.

[ref022] Team R. C 2014 A language and environment for statistical computing. R Foundation for Statistical Computing, Vienna, Austria. URL: (https://www. R-project. org).

[ref024] Vandesompele J., De Preter K., Pattyn F., Poppe B., Van Roy N., De Paepe A. and Speleman F. 2002 Accurate normalization of real-time quantitative RT-PCR data by geometric averaging of multiple internal control genes. Genome Biology 3 (7):research0034-1. 12184808 10.1186/gb-2002-3-7-research0034 PMC126239

